# Coagulen

**Published:** 1915-03

**Authors:** 


**Coagulen**, a new styptic has been "invented" by Theodor Kocher and A. Fonce of Berne. It is said to stop haemorrhage from even large wounds and it may be applied by soldiers in an emergency. Large quantities have been presented to both armies. We have seen no statement of its nature.

